# Curcumin Treatment in Combination with Glucose Restriction Inhibits Intracellular Alkalinization and Tumor Growth in Hepatoma Cells

**DOI:** 10.3390/ijms20102375

**Published:** 2019-05-14

**Authors:** So Won Kim, Min-Ji Cha, Seul-Ki Lee, Byeong-Wook Song, Xinghai Jin, Jae Myun Lee, Jeon Han Park, Jong Doo Lee

**Affiliations:** 1 Department of Pharmacology, Catholic Kwandong University College of Medicine, Gangneung 25601, Korea; kswlab2015@gmail.com; 2 Institute for Translational and Clinical Research, Catholic Kwandong University International St. Mary’s Hospital, Incheon 22711, Korea; minjicha619@gmail.com; 3 KANT Science Research Institute, Incheon 22711, Korea; seulki1011@nate.com (S.-K.L.); jxh630@gmail.com (X.J.); 4 Department of Medical Science, Catholic Kwandong University College of Medicine, Gangneung 25601, Korea; songbw@gmail.com; 5 Brain Korea 21 PLUS Project for Medical Sciences, Yonsei University College of Medicine, Seoul 03722, Korea; jaemyun@yuhs.ac; 6 Department of Microbiology and Immunology, Institute for Immunology and Immunological Diseases, Yonsei University College of Medicine, Seoul 03722, Korea; 7 Department of Nuclear Medicine, Catholic Kwandong University International St. Mary’s Hospital, Incheon 22711, Korea

**Keywords:** hepatoma, intracellular pH, curcumin, glucose restriction, tumor suppression

## Abstract

Dysregulation of cellular energy metabolism is closely linked to cancer development and progression. Calorie or glucose restriction (CR or GR) inhibits energy-dependent pathways, including IGF-1/PI3K/Akt/mTOR, in cancer cells. However, alterations in proton dynamics and reversal of the pH gradient across the cell membrane, which results in intracellular alkalinization and extracellular acidification in cancer tissues, have emerged as important etiopathogenic factors. We measured glucose, lactate, and ATP production after GR, plant-derived CR-mimetic curcumin treatment, and curcumin plus GR in human hepatoma cells. Intracellular pH regulatory effects, in particular, protein–protein interactions within mTOR complex-1 and its structural change, were investigated. Curcumin treatment or GR mildly inhibited Na+/H+ exchanger-1 (NHE1). vATPase, monocarboxylate transporter (MCT)-1, and MCT4 level. Combination treatment with curcumin and GR further enhanced the inhibitory effects on these transporters and proton-extruding enzymes, with intracellular pH reduction. ATP and lactate production decreased according to pH change. Modeling of mTOR protein revealed structural changes upon treatments, and curcumin plus GR decreased binding of Raptor and GβL to mTOR, as well as of Rag A and Rag B to Raptor. Consequently, 4EBP1 phosphorylation was decreased and cell migration and proliferation were inhibited in a pH-dependent manner. Autophagy was increased by curcumin plus GR. In conclusion, curcumin treatment combined with GR may be a useful supportive approach for preventing intracellular alkalinization and cancer progression.

## 1. Introduction

Dysregulation of cellular energy metabolism, known as the Warburg effect, is closely linked to cancer development and progression. Inhibition of enhanced glycolytic activity in cancer cells via calorie restriction (CR) or glucose restriction (GR) is under clinical investigation as a supportive anticancer therapy. CR inhibits energy-dependent signaling pathways, including the insulin-like growth factor 1 (IGF-1)/phosphoinositide 3-kinase (PI3K)/Akt/mammalian target of rapamycin (mTOR) pathway, and activates AMP-dependent protein kinase (AMPK) activity [1].

Recently, alterations in proton dynamics have emerged as an important factor contributing to the etiopathogenesis of cancer cells, and energy dysregulation is thought to be attributable to increased intracellular pH (pHi) [2]. In fact, intracellular alkalinization and extracellular acidification are commonly observed in malignant tumor tissues. This occurs as a result of the export of excess intracellular lactate and protons into the extracellular space by the transmembrane monocarboxylate transporter-4 (MCT4), coupled with the secretion of protons by Na^+^/H^+^ exchanger-1 (NHE1) or proton-extruding enzymes, including vacuolar H^+^-ATPase (v-ATPase) and carbonic anhydrases. Altered activities of transporters or enzymes in cancer cells, and reversal of the pH gradient across the cancer cell membrane, play pivotal roles in cancer progression and metastasis [3,4]. A decrease in the extracellular pH (pHe) stimulates angiogenesis, activates stromal cells for tumor invasion, and impairs the immune reaction as a result of decreased cytolytic functions of T cells [5]. Resistance to chemotherapeutics is also related to increased extracellular acidity [6]. Intracellular alkalinization has important biological effects, and even small changes in the pHi significantly affect protein activities. Therefore, inhibition of intracellular alkalinization is a potential anticancer approach, and various NHE1 inhibitors, such as cariporide, are under investigation [7].

GR decreases lactate production in cancer cells [8], which suggests that energy restriction might be an alternative approach for preventing intracellular alkalinization. GR lowers intracellular ATP concentrations and decreases the pHi [9]. Curcumin prevents the extrusion of intracellular protons by functioning as an NHE1 inhibitor [10]. Furthermore, the pHi affects the conformation and binding affinity of various proteins [11].

As we have previously demonstrated that GR combined with CR-mimetic plant-derived polyphenols has synergistic anticancer effects in malignant tumors [12], we investigated the anti-cancer effects of GR or curcumin treatments, and especially combination treatment in terms of intracellular pH regulation in human hepatoma cell lines because glucose metabolism is closely linked to cytosolic pH regulation. Furthermore, protein–protein interactions within mTOR complex-1 (mTORC1) following curcumin and/or GR treatment were studied as mTORC1 plays critical roles in cell size regulation, cell growth, and nutrient sensing. 

## 2. Results

### 2.1. Curcumin and GR Inhibit Intracellular Alkalinization and Function as NHE1 Inhibitors

**Figure 1:**
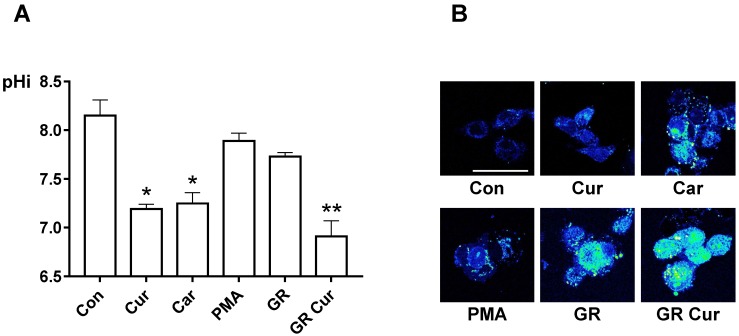
pHi-lowering effect of curcumin and glucose restriction. (**A**) HepG2 cells were cultivated with standard medium, standard medium containing 20 nM curcumin, 100 nM cariporide, or 100 nM PMA, GR (5.5 mM), or GR containing 20 nM curcumin, then pHi was measured. The experiment was conducted five times independently. (**B**) pHi imaging was performed using confocal microscopy (400×). Bright green color and dark blue color indicate acidic and alkaline condition, respectively. The scale bar is 50 μm. Con, standard RPMI-1640 medium; Cur, curcumin; Car, cariporide; PMA, phorbol-12-myristate-13-acetate; GR, glucose restriction, 5.5 mM glucose medium; GR Cur, glucose restriction plus curcumin. * *p* < 0.05 vs. control; ** *p* < 0.01 vs. control.

Enhanced aerobic glycolysis (Warburg effect) is one of the hallmarks of cancer cells. Because glycolysis is closely linked to the cytosolic pH and export of H^+^ ions, whether curcumin and/or GR affect the pHi was investigated in the hepatoma cell lines HepG2, Hep3B, and SNU449. The pHi in these cell lines was decreased when cells were cultured with standard RPMI-1640 medium (11 mM glucose) with curcumin, low glucose concentration (5.5 mM, GR), and more decreased under GR plus curcumin as compared to standard medium (11 mM glucose) without curcumin. Treatment effects were more prominent in HepG2 cells (Supplementary Figure S1). The pHi of HepG2 cells grown in the standard medium was 8.15 ± 0.16, and it was significantly decreased after curcumin administration (7.19 ± 0.05, *p* < 0.05), and mildly decreased under GR condition (7.73 ± 0.04, *p* > 0.05). Curcumin administration under GR condition decreased the pHi to a lower normal limit (6.91 ± 0.16, *p* < 0.01). Curcumin inhibited intracellular alkalinization as effectively as the NHE1 inhibitor, cariporide (7.25 ± 0.11, *p* < 0.05). However, the NHE1 activator PMA did not significantly increase the pHi (7.89 ± 0.08, *p* > 0.05) (Figure 1A). 

Fluorescence visualization of the pHi by BCECF-AM confirmed that curcumin decreased the pHi similar to cariporide, and combination of curcumin with GR resulted in more effective pHi suppression on fluorescent imaging (Figure 1B). However, the pHi of human dermal fibroblast cells was within the normal range after GR plus curcumin (Supplementary Figure S2).

### 2.2. Curcumin and GR Inhibit Level of Proton-Extruding Proteins

**Figure 2:**
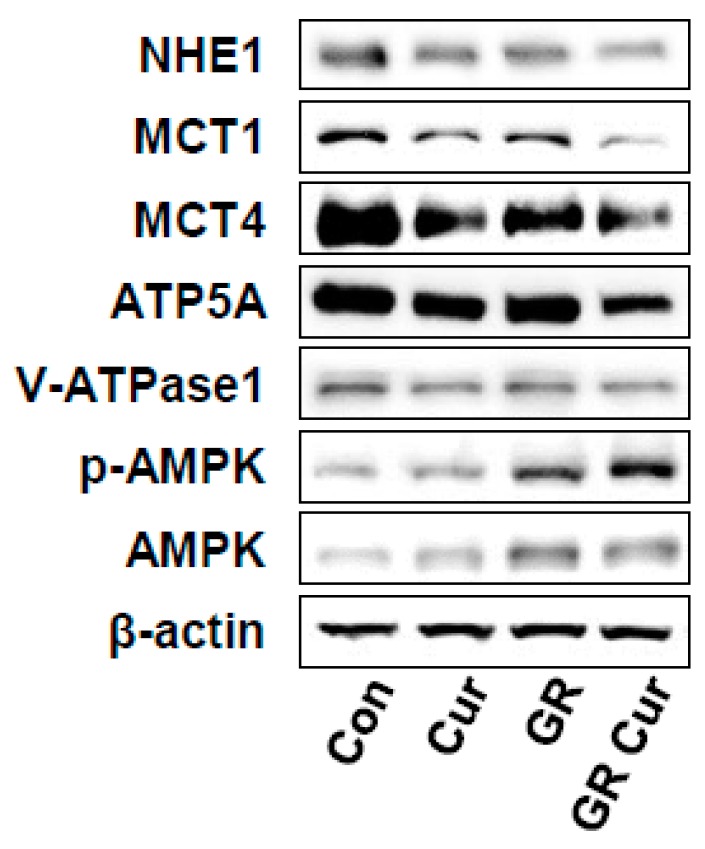
Effect of curcumin and glucose restriction on the protein level of transporters, enzymes regulating pHi, and the energy regulator AMPK. HepG2 cells were cultivated under the conditions indicated in the legend of Figure 1, and immunoblotting was performed using appropriate antibodies to NHE1, MCT1, MCT4, ATP5A, v-ATPase1, p-AMPK, and AMPK, respectively. β-Actin was used as a loading control. The experiment was conducted three times independently. Con, standard RPMI-1640 medium; Cur, curcumin; GR, glucose restriction, 5.5 mM glucose medium; GR Cur, glucose restriction plus curcumin.

To elucidate the pHi regulatory mechanisms of curcumin and GR, the effect of curcumin and GR on the level of the proton-extruding proteins NHE1, MCTs, and v-ATPase was investigated in HepG2 cells by immunoblotting. Protein level of NHE1 was decreased in HepG2 cells grown in standard medium with curcumin, or GR alone, and these effects were more prominent in the GR plus curcumin group (Figure 2). Protein level of MCT1 and MCT4 was also significantly decreased under the treatment conditions. ATP synthase (ATP subunit alpha, ATP5A) and v-ATPase were decreased under the same treatment conditions (Figure 2). These findings indicated that the level changes of these proteins by curcumin and GR were correlated with pHi changes. Thus, curcumin and GR might in part regulate pHi by modulating the level of proton-extruding proteins. Curcumin suppressed NHE1 mRNA to the same level as cariporide. Upon treatment with PMA, the mRNA level of NHE1 was slightly increased. Combination of GR and curcumin reduced the mRNA level of NHE1 the most significantly (Supplementary Figure S3). In contrast, AMPK and p-AMPK were markedly increased under GR conditions (>3-fold increases, *p* < 0.01) (Figure 2).

### 2.3. Glucose Uptake and Lactate Production are Affected by pHi, and Inhibited by Curcumin and GR

**Figure 3:**
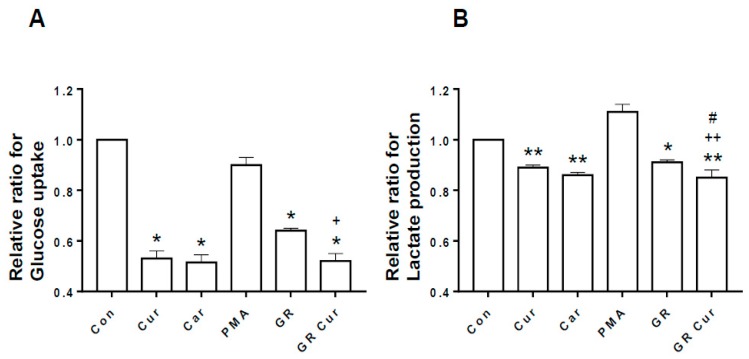
Effect of curcumin and glucose restriction on glucose uptake and lactate production. HepG2 cells were cultivated under the conditions indicated in the legend of Figure 1, and (**A**) glucose uptake and (**B**) lactate production were quantified. Con, standard RPMI-1640 medium; Cur, curcumin; Car, cariporide; PMA, phorbol-12-myristate-13-acetate; GR, glucose restriction, 5.5 mM glucose medium; GR Cur, glucose restriction plus curcumin. Experiment was conducted five times independently. * *p* < 0.05 vs. control; ** *p* < 0.01 vs. control; + *p* < 0.05 vs. GR; ++ *p* < 0.01 vs. GR; # *p* < 0.05 vs. Cur.

Because enhanced glucose uptake and enhanced lactate formation are key features of cancer cells, whether changes of pHi by curcumin and GR affect glucose uptake and lactate formation was investigated in HepG2 cells. Glucose uptake was significantly decreased after treatment with curcumin, and/or GR as NHE-1 inhibitor cariporide when compared with the control (Figure 3A). Lactate production was mildly decreased after treatment with curcumin, cariporide, and/or GR (Figure 3B). Therefore, glucose uptake and lactate production appear to be associated with pHi changes by curcumin and GR, although curcumin did not produce synergic effect with GR.

### 2.4. Intracellular ATP is Linked to pHi Change

**Figure 4:**
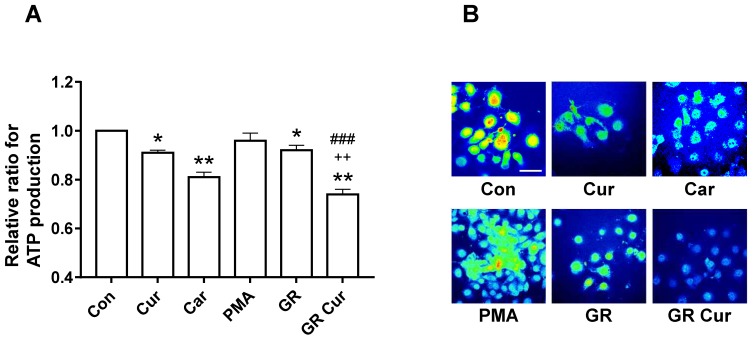
Effect of curcumin and glucose restriction on ATP production. HepG2 cells were cultivated under the conditions indicated in the legend of Figure 1, and (**A**) ATP production was measured. Experiment was conducted five times independently. (**B**) Confocal imaging of ATP production in HepG2 cells. High and low ATP concentrations are indicated by bright red and dark blue color, respectively. The scale bar is 50 μm. Con, standard RPMI-1640 medium; Cur, curcumin; Car, cariporide; PMA, phorbol-12-myristate-13-acetate; GR, glucose restriction, 5.5 mM glucose medium; GR Cur, glucose restriction plus curcumin. * *p* < 0.05 vs control; ** *p* < 0.01 vs. control; ++ *p* < 0.01 vs. GR; ### *p* < 0.005 vs. Cur.

Next, ATP production in HepG2 cells treated with curcumin and GR was assessed. ATP production was mildly decreased after curcumin treatment or GR alone. It was significantly further decreased after curcumin treatment in a GR condition (Figure 4A). Confocal imaging of intracellular ATP confirmed these results with a marked decrease in ATP, and smaller cell size after curcumin treatment in a GR compared to cells grown in the standard medium (Figure 4B). ATP concentrations were also decreased in Hep3B and SNU449 cells after curcumin treatment in a GR condition (Supplementary Figure S4A,B). These findings suggested that ATP production is controlled by pHi changes due to curcumin and GR. No significant treatment effects on ATP production were observed in human melanocytes and dermal fibroblasts (Supplementary Figure S4C,D).

### 2.5. Curcumin and GR Induce Structural Changes in the mTOR Protein and Changes in the Binding of mTORC1 Interacting Proteins by Affecting pHi

**Figure 5:**
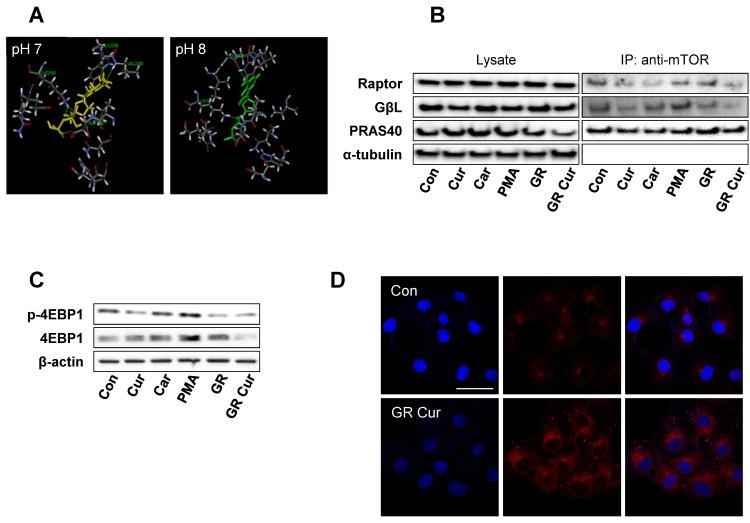
pH-dependent structural changes and alteration of mTORC1 interaction degree according to pHi changes induced by curcumin and glucose restriction. (**A**) Structural changes of the mTOR-GβL complex at the ATP-binding site (PDB code: 4JSV) were analyzed by computational modeling using the Pipeline Pilot 8.5. In the original structure at pH 8, the activity-site cavity size is 7.08 A, whereas at pH 7, the activity-site cavity size is 8.09 A. The ATP-binding site is indicated by yellow (pH 7) and green (pH 8) solid lines, respectively. Green dashed lines indicate hydrogen bonds. The names of residues involved in hydrogen bonding are indicated. (**B**) Using HepG2 cells, co-immunoprecipitation was used to detect mTOR interacting proteins. The experiment was conducted three times independently. (**C**) For the determination of mTOR activity, the mTOR downstream signal phosphor-4EBP1 was detected by immunoblotting. β-Actin was used as a loading control. The experiment was conducted three times independently. (**D**) Confocal imaging of autophagosomes. Cells were immunostained with an LC3 antibody. The scale bar is 50 μm. Con, standard RPMI-1640 medium; Cur, curcumin; Car, cariporide; PMA, phorbol-12-myristate-13-acetate; GR, glucose restriction, 5.5 mM glucose medium; GR Cur, glucose restriction plus curcumin. * *p* < 0.05 vs. control.

To compare the original structure of mTOR protein crystallized at pH 8, ionization of the mTOR-GβL protein-binding site at pH 7 was evaluated by computational analysis using Pipeline Pilot 8.5. The pattern and total numbers of hydrogen bonds between ATP and mTOR protein were changed, and the active binding cavity size increased from 7.08 A to 8.09 A (Figure 5A). 

Because GR alters energy-dependent signaling pathways, such as the IGF-1-PI3K-Akt-mTOR pathway [1], and pHi affects the conformation and binding affinities of various proteins [11], mTOR co-IP and Raptor co-IP were carried out. Co-IP of the mTOR protein showed a decrease in binding of both GβL and Raptor to mTOR after curcumin and/or GR treatment as cariporide, especially GβL binding was more significantly decreased by GR plus curcumin. However, PRAS40 binding to mTOR was not significantly reduced (Figure 5B). Consequently, phosphorylation of eukaryotic translation initiation factor 4E-binding protein 1 (p-4EBP1), one of the downstream targets of mTOR [13], was decreased upon treatment with curcumin, cariporide, or GR. In accordance with the pHi pattern, phosphorylation of 4EBP1 was the most strongly decreased by GR plus curcumin treatment (Figure 5C). Consequently, autophagy was remarkably induced after curcumin treatment under GR condition in both HepG2 and Hep3B cells (Figure 5D and Supplementary Figure S5A). In human dermal fibroblasts, the treatments had no significant effects (Supplementary Figure S5B). 

### 2.6. GR Plus Curcumin Treatment Results in Diminished Cell Migration, Growth Inhibition, and Apoptosis

**Figure 6:**
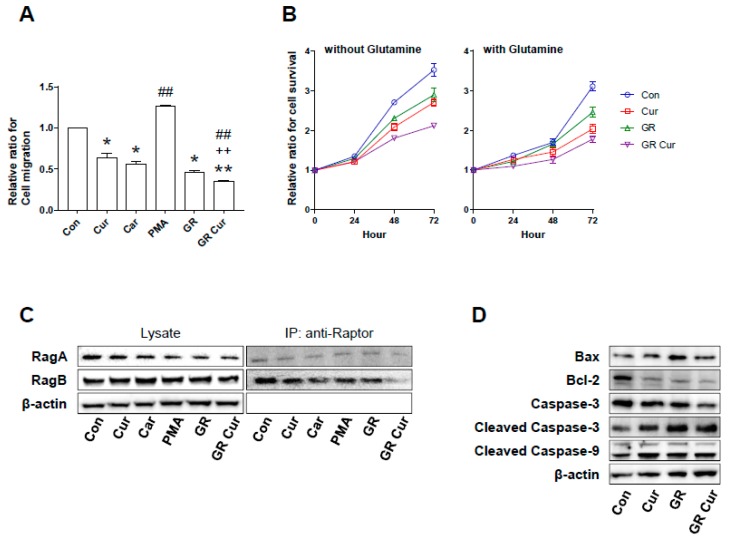
Effect of curcumin and glucose restriction on cell migration, proliferation, and death in HepG2 cells. (**A**) Transwell migration of HepG2 cells. Results were obtained from three independent experiments, and bar graphs represent the cell number per image field (mean ± SE). The experiment was conducted five times independently. (**B**) Cell proliferation rates of HepG2 cells were measured using a Cell Counting Kit-8 (CCK-8). The presence or absence of glutamine was observed. The experiment was conducted five times independently. (**C**) Co-immunoprecipitation was used to detect RagA and RagB. The experiment was conducted three times independently. (**D**) Bax, Bcl-2, Caspase-3 and -9, cleaved Caspase-3 and -9 proteins were expressed by immunoblotting. Beta actin was used as an internal control. The experiment was conducted three times independently. Con, standard RPMI-1640 medium; Cur, curcumin; Car, cariporide; PMA, phorbol-12-myristate-13-acetate; GR, glucose restriction, 5.5 mM glucose medium; GR Cur, glucose restriction plus curcumin. * *p* < 0.05 vs. control; ** *p* < 0.01 vs. control; ++ *p* < 0.01 vs. GR; ## *p* < 0.01 vs. Cur.

Migration and proliferation are malignant phenotypes of cancer cells. Therefore, the migration and proliferation potential of HepG2 cells treated with curcumin and/or GR were assessed. Significantly attenuated migration potential was observed in HepG2 cells treated with curcumin and/or GR when compared to cells cultivated in standard medium (Figure 6A). The cell migration rate was found to be related with pHi changes induced by curcumin and/or GR. GR plus curcumin led to a significant reduction in cell proliferation potential of HepG2 cells. Glutamine addition in the culture media by 2 mM did not increase cell proliferation (Figure 6B). IP experiments conducted using Raptor antibody showed that the RagA and RagB proteins did not achieve good complex with raptor under CR+Cur condition (Figure 6C). 

Bax protein level was increased, and protein level of Bcl-2, a negative regulator of Bax, was decreased with curcumin and/or GR. Caspase-3 protein level was suppressed by curcumin and/or GR treatment, whereas cleaved caspase-3 was increased by curcumin and/or GR treatment. Cleaved caspase-9 protein level was similar to that of cleaved caspase-3 (Figure 6D). Thus, curcumin and/or GR suppressed Bcl-2 protein level and induced Bax protein level and cleavage of Caspase-3 and -9, which indicates activation of apoptotic pathways.

## 3. Discussion

Reversal of the pH gradient across the cell membrane with intracellular alkalinization and extracellular acidification is a hallmark of cancer cells. In healthy cells, the pHi is approximately 7.2 and the pHe is 7.4. In tumor cells, the pHi is >7.2, whereas the pHe decreases to 6.7–7.1 [3]. Reversal of the pH gradient has important effects on cell physiology. Protonation or deprotonation significantly affect the charge status of many amino acid side chains, resulting in post-translational modifications of various proteins via phosphorylation, acetylation, and ubiquitination. It may disrupt protein–protein binding affinities, localization of proteins on the cell membrane, and the assembly of macromolecular subunits [14,15]. Limited increases in pHi, especially alkaline pHi, have important advantages for cell growth [3], inhibit apoptosis [16], and enhance glycolytic enzyme activity [17]. However, acidification of the extracellular microenvironment potentiates tumor invasion and metastasis.

Previous studies have demonstrated that intracellular alkalinization is the primary transforming event in cancer cells. Transfection of a yeast proton-pumping ATPase into mouse NIH3T3 cells or monkey Vero fibroblasts resulted in intracellular alkalinization with malignant transformation. Thus, yeast ATPase gene behaves as an oncogene [18]. Another study revealed that NIH3T3 cells infected with recombinant retroviruses expressing E7 oncogene of the human papilloma virus type 16 displayed cytoplasmic alkalinization driven by NHE1 activation. Subsequently, glycolytic activity was increased [2]. Conversely, NHE1 inhibition diminishes tumorigenic potential [19], and acidification of the cytoplasm leads to apoptosis [20]. Therefore, targeting proton dynamics associated with the intracellular pH gradient has been proposed as a potential cancer prevention strategy and therapeutic approach.

This study revealed that curcumin plus GR lowered the pHi in several hepatoma cell lines as effectively as the NHE1 inhibitor cariporide. Among the hepatoma cell lines, HepG2 cells were the most significantly affected, although GR alone could mildly inhibit NHE1. As NHE1 activation is energy- and Akt-dependent [21,22]. GR appears to potentiate the ability of curcumin to inhibit NHE1 synergistically, and vice versa. 

The exact mechanism by which curcumin inhibits NHE1 needs to be elucidated. However, studies have demonstrated that curcumin affects various ion channels and transporters [23] and intracellular proton extrusion [10]. In this study, both mRNA and protein levels of NHE1 were decreased by curcumin treatment regardless of GR. This suggests that pHi alterations might affect both transcription and translation of various proteins, as previously reported [24]. In addition, proton-extruding enzymes, including v-ATPase and ATP synthase, were also decreased in our study. This is consistent with a previous report that v-ATPase can be inhibited by GR through disruption of v-ATPase assembly [9].

MCT4 regulates pHi by exporting excess lactate and protons from the cytoplasm of cancer cells. Lactate in the extracellular space affects cancer development and progression by acidifying the cellular microenvironment and by inducing the secretion of cytokines and growth factors needed for tumor growth. When lactate is extruded from hypoxic cancer cells by MCT4, it can be imported into less hypoxic cancer cells, stromal cells, or vascular endothelial cells by MCT1 as an energy metabolite [25]. Imported lactate activates hypoxia inducible factor-1 (HIF-1) through stabilization of the HIF-1α subunit, independently of hypoxia [26]. Therefore, MCT4 and MCT1 form a lactate shuttle between cancer cells and stromal cells (reverse Warburg effect), hypoxic cancer cells, and oxidative cancer cells (metabolic symbiosis), as well as between hypoxic cancer cells and vascular endothelial cells [27,28]. This provides the energy required for tumor growth [29]. In this study, the protein level of both MCT1 and MCT4 was decreased by curcumin treatment in a low-glucose condition. This suggests that the curcumin plus GR might restrain not only hypoxic cancer cells, but also oxidative cancer cells, stromal, and vascular endothelial cells.

In this study, glucose uptake and lactate formation were decreased by curcumin plus GR. These findings suggest that low glucose uptake and low lactate production were associated with the pHi changes induced by curcumin and GR. The intracellular ATP concentration was decreased by 26% in HepG2 cells upon curcumin treatment under low-glucose condition (*p* < 0.01). This was a more profound reduction than that induced by cariporide treatment. In contrast, curcumin treatment or low-glucose condition alone induced only a mild reduction. The dynamics of intracellular ATP concentrations appear to correlate with changes in pHi and lactate production. Although curcumin treatment alone can inhibit ATP synthase [30], the low-glucose condition restricted the energy supply needed for intracellular ATP synthesis (Figure 4). Finally, growth inhibition and cell death of cancer cells was induced by treatment with curcumin under low-glucose condition, likely through a decline in ATP synthesis and a decrease in pHi [31,32]. Therefore, curcumin plus GR might be an effective anticancer approach by reducing the pHi. Additionally, this treatment strategy might prevent chemoresistance because high intracellular ATP levels are associated with chemoresistance, particularly in colon cancer cells [33].

Intriguingly, the protein levels of AMPK and p-AMPK significantly increased in the low-glucose condition, possibly as a result of diminished glucose availability and low intracellular ATP levels. However, it is also possible that curcumin might mildly activate AMPK [34,35,36]. Therefore, GR would synergistically enhance the anticancer activity of pHi-lowering agents because GR-induced AMPK activation enhances NHE1 inhibitor activity [37].

Next, structural changes in mTOR protein and pH-dependent protein–protein interactions required for mTORC1 assembly were investigated using computer modeling and co-IP, respectively. It is well known that mTOR forms a complex, especially with Raptor [38] and GβL [39]. Computer modeling of mTOR protein showed that the mTOR structure is affected by pHi, and mTOR binding with GβL and Raptor was significantly diminished by curcumin treatment under low-glucose condition. This was correlated with the reduction in pHi. Consequently, downstream 4EBP1 phosphorylation was decreased, because GβL is a positive regulator of mTOR kinase activity in conjunction with Raptor [39]. In addition, hepatoma cells were smaller, with intensely stained autophagosomes, especially under curcumin treatment plus GR. mTOR is well known as a regulator of autophagy. mTOR basically is an inhibitor to autophagosome formation. PI3K and AKT control mTOR, and under nutrient starvation, mTOR induces autophagy [40,41]. Accordingly, in this study, autophagy was observed in the starvation condition, and the addition of curcumin further enhances autophagy induction. GR and curcumin treatment reduced the intracellular pH, which might have altered the structure of mTOR and consequently weakened interactions with interactors such as Raptor, GβL, which together form mTORC1.

In cancer cell metabolism, amino acids are as important as glucose as an energy source. mTORC1 plays a pivotal role in amino acid-dependent cell proliferation. Therefore, it needs to be elucidated whether high concentrations of amino acids attenuate the anticancer effects of GR. During amino acid-dependent mTORC1 activation, Ras-related small guanosine triphosphate (GTP)-binding proteins, RagA or RagB, bind to RagC or RagD to form heterodimers. These heterodimers actively bind to Raptor [42], which recruits mTORC1 to the surface of the lysosomal membrane [43]. In addition, v-ATPase is necessary for lysosome-associated mTORC1 activation [44]. However, our data demonstrated that Raptor binding to mTOR, RagA, and RagB, and v-ATPase protein level were all diminished after curcumin treatment under GR. Furthermore, amino acids such as leucine (data not shown) or glutamine supplementation did not affect cell growth. These findings imply that in clinical practice, protein restriction—e.g., through ketogenic diet—may not be necessary. 

Reducing cytoplasmic alkalinization might significantly affect the conformation of various proteins [9], which in turn affects post-translational modifications of other proteins in a chain reaction, leading to tumor cell death or delayed cell growth. Additionally, polymerization and remodeling of the actin filaments that regulate cancer cell migration [4,11,45] can be inhibited. Likewise, HepG2 cell migration was significantly inhibited by curcumin or GR, and most effectively by curcumin plus GR. These effects were observed to be pHi-dependent.

Curcumin has long been reported to have a problem with bioavailability such as low serum levels and limited tissue distribution due to its poor absorption and rapid metabolism [46]. For these reasons, various strategies are used to enhance bioavailability through innovative drug delivery systems such as liposome, nanoparticle, phospholipid encapsulation, and developing new curcumin analogues [47,48,49]. Currently, products that enhance bioavailability are being sold, and it is thought curcumin products will continue to be released in the future [49].

## 4. Materials and Methods

### 4.1. Chemicals and Antibodies

Cariporide, curcumin, and phorbol-12-myristate-13-acetate (PMA) were purchased from Sigma-Aldrich (St. Louis, MO, USA). Antibodies against NHE1, MCT1, MCT4, ATP5A, v-ATPase, β-actin, α-tubulin, Rag-A, Rag-B, Raptor, Caspase 3, Caspase 9, cleaved Caspase 3, and cleaved Caspase 9 were purchased from Santa Cruz Biotechnology (Dallas, TX, USA). Antibodies against AMPK, p-AMPK, GβL, PRAS40, and p-4EBP1 were purchased from Cell Signaling Technology (Danvers, MA, USA).

### 4.2. Cell Lines and Cell Culture

HepG2 and Hep3B human hepatocellular carcinoma cell lines were obtained from the American Type Culture Collection (ATCC, Manassas, VA, USA). The HDFa normal human dermal fibroblast line and HEMa normal human epidermal melanocyte line were obtained from the ATCC. The human hepatoma cell line SNU449 was obtained from the Korean Cell Line Bank (Seoul, Korea). All cells were maintained in RPMI-1640 medium (Welgene, Gyeongsan, Korea) supplemented with 10% fetal bovine serum (FBS) (HyClon, Tauranga, New Zealand), 100 U/mL penicillin, and 100 µg/mL streptomycin (HyClone). The cells were maintained at 37 °C in a 5% CO_2_ humidified incubator. For growing cells at high glucose concentration, standard RPMI-1640 medium was used. For growing cells at low glucose concentration, a mixture (1:1, *v*/*v*) of standard and glucose-free RPMI-1640 medium was used.

### 4.3. Reverse Transcriptase Polymerase Chain Reaction (RT-PCR)

Total RNA was isolated from HepG2 cells using TRIZOL Reagent (Takara, Kusatsu, Shiga, Japan) and the RNA concentration was measured spectrophotometrically (Gen5TM) (BioTek, Winooski, VT, USA). RNA (2 μg) was reverse-transcribed using 5× PrimeScript RT Master Mix (Takara, Japan). The cDNA was used as a template for PCR amplification using the following thermal cycles: 95 °C for 5 min, 30 cycles of 95 °C for 30 s, 58 °C for 30 s, and 72 °C for 60 s. PCR products were analyzed on a 1% agarose gel. PCR primer sequences were as follows: NHE1, 5′-TCT TCA CCG TCT TTG TGC AG-3′ and 5′-CTT GTC CTT CCA GTG GTG GT-3′; GAPDH, 5′-CTC ATG ACC ACA GTC CAT GCC ATC-3′ and 5′-CTG CTT CAC CAC CTT CTT GAT GTC-3′.

### 4.4. Real-Time (q)RT-PCR

RNA was extracted from cells using TRIZOL reagent (Invitrogen, Carlsbad, CA, USA) and reverse-transcribed using random hexamers and SuperScript IV Reverse Transcriptase (Invitrogen). cDNA was used for qPCR with TaqMan Universal Master Mix II (Applied Biosystems, Foster City, CA, USA). Taqman primers used were: NHE1 (SLC9A1): Hs00300047_m1, GAPDH: Hs03929097_g1. Thermal cycles were: 95 °C for 20 s, 40 cycles of 95 °C for 3 s and 60 °C for 30 s, and finally, 95 °C for 15 s, 60 °C for 1 min, and 95 °C for 15 s. Relative level was analyzed according to the comparative cycle threshold (Ct) method and was normalized to the mRNA level of *GAPDH* in each sample [50]. *NHE1* mRNA level was examined in triplicate and the experiment was repeated at least three times. 

### 4.5. Immunoblotting

After harvesting cells by centrifugation, pellets were resuspended in 100 µL of lysis buffer (20 mM HEPES, (pH 7.5), 1.5 mM MgCl_2_, 10 mM KCl, 1 mM EDTA, 1 mM EGTA, 250 mM sucrose, 0.1 mM PMSF, 1 mM dithiothreitol, 4 μg/mL pepstatin, 4 μg/mL leupeptin, and 5 μg/mL aprotinin). After incubation on ice for 10 min, the cells were centrifuged at 750× *g* at 4 °C for 10 min. The supernatant was collected and centrifuged at 10,000× *g* for 10 min at 4 °C. Protein concentrations were determined using bicinchoninic acid (Thermo Fisher Scientific, Waltham, MA, USA). Proteins were separated by sodium dodecyl sulfate–polyacrylamide gel electrophoresis (SDS-PAGE) using 8–15% polyacrylamide gels and then electrotransferred to methanol-treated polyvinylidene difluoride membranes. The membranes were blocked with Tris-buffered saline with 0.1% Tween 20 (TBS-T) containing 5% fat-free powdered milk at room temperature for 1 h. Then, they were washed twice with TBS-T and incubated with primary antibodies at room temperature for 1 h or overnight. The membranes were washed three times with TBS-T for 10 min and then incubated with horseradish peroxidase-conjugated secondary antibodies at room temperature for 1 h. After thorough washing, protein bands were detected using chemiluminogenic reagent (GE Healthcare Life Sciences, Marlborough, MA, USA). 

### 4.6. pHi Measurement 

Cells were cultured on coverslips with 10% standard RPMI-1640 medium containing 11 mM glucose (control), RPMI-1640 containing 5.5 mM glucose (GR), standard RPMI-1640 medium with 20 nM curcumin dissolved in dimethylsulfoxide, or low-glucose medium with curcumin. The concentration of curcumin may vary depending on the cells and experimental conditions. Experiments were conducted at various concentrations and changed in physiological phenomenon were observed at 20 nM. In addition, HepG2 cells were incubated with 100 nM cariporide (an NHE1 inhibitor) or 100 nM PMA (an NHE1 activator) for 30 min. Then, the cells were washed with cold phosphate-buffered saline (PBS; 137 mM NaCl, 2.7 mM KCl, 10 mM Na_2_HPO_4_, 2 mM KH_2_ PO_4_). The cells were incubated with 1.5 mg/L of the fluorescent pH probe 2′,7′-bis-(2-carboxyethyl)-5-(and-6)-carboxyfluorescein acetoxymethyl ester (BCECF-AM; Thermo Fisher Scientific) for 10 min. The cells were resuspended in gradient pH calibration solution (4 mM KOH, 2 mM H3PO2, 135 mM KCl, 20 mM HEPES, 1.2 mM CaCl2, 0.8 mM MgSO4). Read fluorescence intensity was determined by fluorescence-assisted cell sorting using the FL-1 channel (Accuri C6 plus; BD Biosciences, Franklin Lakes, NJ, USA).

### 4.7. Glucose Uptake Measurement

Uptake of 2-deoxyglucose was estimated by an enzymatic NADPH-amplifying system. Briefly, HepG2 cells were seeded into 4-well plates and incubated overnight. Then, the cells were incubated with each culture medium at 37 °C for 12 h. The cells were washed three times with PBS, and lyzed to prepare a 50-μL reaction system. Cell lysates were diluted 1:1, and HepG2 cells were diluted 1:10. After a series of reactions, samples were analyzed spectrophotometrically at excitation and emission wavelengths of 535 nm and 587 nm, respectively. A standard curve was generated by placing 2-deoxyglucose-6-phosphate standard solutions in the wells of a culture plate that had been prepared without cells. Cells were allowed to rest for at least 5 min before measurements.

### 4.8. Lactate Assay

From each well of the 24-well plates, 25 μL of medium was collected and mixed with 100 μL of NADH solution (0.03% β-NAD in the reduced form of disodium salt in phosphate buffer) and 25 μL of pyruvate solution (22.7 mM pyruvic acid in phosphate buffer) at room temperature. NADH consumption was quantified for 2 min by measuring the absorbance at 340 nm. The mean absorbance change (ΔA/min) was calculated for each treatment condition and was expressed as a percentage of the level from cells (100%) induced by 500 μM NMDA. Each treatment was tested in four replicate wells, and the experiment was repeated five to six times.

### 4.9. ATP Assay 

Intracellular ATP was analyzed using an ATP Fluorometric Assay Kit (Abcam, Cambridge, MA, USA), according to the manufacturer’s instructions. HepG2 cells were cultured on coverslips with 10% standard RPMI-1640 medium containing 11 mM glucose (control), RPMI-1640 containing 5.5 mM glucose (GR), standard RPMI-1640 medium with 20 nM curcumin dissolved in dimethylsulfoxide, or low-glucose medium with curcumin. In addition, HepG2 cells were incubated with 100 nM cariporide, or 100 nM PMA for 12 h. In total, 1 × 10^6^ cells were washed twice with cold PBS and resuspended in 100 µL of ATP assay buffer. A 50-µL volume of cell suspension (0.5 × 10^6^ cells) was mixed with 2 µL of ATP probe. After the mixture was gently vortexed, the cells were incubated in the dark at room temperature for 30 min and then subjected to immunofluorescence analysis using a microplate reader (Varioskan Flash; Thermo Fisher Scientific, Waltham, MA, USA). The ATP assay protocol relies on the phosphorylation of glycerol to generate a product that is easily quantified by fluorometry (Ex/Em = 535/587 nm).

### 4.10. In Silico Analysis of mTOR Protein Structure

Structural changes of mTOR-GβL complex at the ATP-binding site (PDB code: 4JSV) [51] were analyzed using Pipeline Pilot 8.5 (Biovia, San Diego, CA, USA). Chemistry at Harvard Macromolecular Mechanics (CHARMm) force-field was assigned to the structure, and the Momany–Rone method [52] was used for partial charge estimation. Parameters were set to default settings. 

### 4.11. Co-Immunoprecipitation (-IP)

Cells were lyzed in IP lysis buffer (Thermo Fisher Scientific) on ice for 30 min. Cell lysates were centrifuged at 10,000× *g* for 20 min, and the supernatant was collected. After protein quantification, the lysates were incubated with antibodies against mTOR, Raptor, GβL, PRAS40, Rag-A, or Rag-B. The tubes were rotated at 4 °C for 1 h. Then, Protein G Dynabeads (Thermo Fisher Scientific) were added to the lysates and incubated overnight at 4 °C. The Dynabeads-antibody-mTOR complexes and antibody-Raptor complexes were centrifuged at 2500× *g* at 4 °C for 30 min, and the antibody-mTOR and antibody-Raptor complexes were eluted with elution buffer. The eluted proteins were heated at 99 °C for 10 min and subjected to SDS-PAGE.

### 4.12. Immunohistochemistry for Autophagy

Cells were cultured in 4-well slide chambers, washed twice with PBS, and fixed in a 1% paraformaldehyde solution for 10 min. The cells were washed twice with PBS prior to permeabilization in 0.1% Triton X-100 for 10 min. Next, the cells were blocked in blocking solution (2% BSA and 10% horse serum in PBS) for 1 h. They were then incubated with LC3 primary antibody (1:100 dilution, Santa Cruz Biotechnology), followed by incubation with FITC-conjugated mouse or Texas Red-conjugated rabbit secondary antibody (Jackson ImmunoResearch Laboratories, West Grove, PA, USA). Nuclei were counterstained with DAPI (Thermo Fisher Scientific) for 30 min. The cells were rinsed six times with PBS to remove excess DAPI. Immunofluorescence was detected by confocal microscopy (LSM780; Carl Zeiss, Oberkochen, Germany).

### 4.13. Cell Viability Assay

Cell viability was determined using a WST-8 (2-[2-methoxy-4-nitrophenyl]-3-[4-nitrophenyl]-5-[2,4-disulfophenyl]-2H-tetrazolium) assay kit (CCK-8 assay kit; Dojindo, Kumamoto, Japan). HepG2 cells were seeded into 96-well plates (Corning Inc., Corning, NY, USA) at 5 × 10^3^ cells/well. After incubation for 24 h, the cells were treated with curcumin (20 nM) in 10% RPMI-1640 containing glucose at different concentrations (11, 5.5, or 2 mM). The cells were washed twice with culture medium, and 100 μL of CCK-8 reagent was added to each well. The plates were incubated at 37 °C for 2 h. Absorbance in each well was measured at 450 nm using a microplate reader (Bio-Rad, Hercules, CA, USA) and corrected for background. L-glutamine concentration was 2 mM.

### 4.14. Cell Migration Assay

Cell migration was assayed using a modified version of the Boyden chamber method [53], which employs microchemotaxis chambers and polycarbonate filters with a pore size of 8.0 μm. Cells were trypsinized and suspended at 5 × 10^5^ cells/mL in culture medium supplemented with 10% FBS. Cell suspension (100 μL) was placed in the upper chamber, and the experimental medium (600 μL/well) was added to the lower chamber. The chamber was incubated at 37 °C under 5% CO_2_ for 48 h. Then, the filter was removed, and cells on the upper side of the filter were scraped off using a cotton tip. Cells migrated to the lower side of the filter were fixed in methanol and stained with hematoxylin. Cells in three randomly selected fields were counted at a magnification of 200× under an inverted microscope. Each treatment was analyzed in triplicate and the experiment was repeated at least three times.

### 4.15. Statistical Analysis

Data are expressed as the means ± standard errors (SEs). Data for two groups were compared using Student’s *t*-test. Comparisons among more than two groups were conducted by one-way analysis of variance (ANOVA) followed by Bonferroni tests. *p* < 0.05 was considered significant.

## 5. Conclusions

In conclusion, curcumin treatment in combination with GR without calorie or protein restriction could be a useful anticancer approach to prevent intracellular alkalinization and inhibit enhanced glycolysis. In clinical practice, this might be a more useful chemoprevention strategy than CR or GR diets, such as a ketogenic diet.

## Supplementary Materials

Supplementary materials can be found at https://www.mdpi.com/1422-0067/20/10/2375/s1. **Supplementary Figure S1.** pHi-lowering effect of curcumin and glucose restriction in the human hepatoma cell lines HepG2, Hep3B, and SNU449. The experiment was conducted five times independently. Con, standard RPMI-1640 medium; Cur, curcumin; GR, glucose restriction, 5.5 mM glucose medium; GR Cur, glucose restriction plus curcumin. * *p* < 0.05 vs. control; ** *p* < 0.01 vs. control. **Supplementary Figure S2.** Effect of curcumin and glucose restriction on the pHi in HDFa human dermal fibroblasts. The pHi is within the normal range, even after curcumin treatment under glucose restriction condition. experiment was conducted five times independently. The experiment was conducted five times independently. Con, standard RPMI-1640 medium; Cur, curcumin; Car, cariporide; PMA, phorbol-12-myristate-13-acetate; GR, glucose restriction, 5.5 mM glucose medium; GR Cur, glucose restriction plus curcumin **Supplementary Figure S3**. NHE1 mRNA level affected by curcumin, cariporide, PMA, and glucose restriction conditions. NHE1 mRNA level was detected by (**A**,**B**) RT-PCR and (**C**) qRT-PCR. The experiment was conducted three times independently. Con, standard RPMI-1640 medium; Cur, curcumin; Car, cariporide; PMA, phorbol-12-myristate-13-acetate; GR, glucose restriction, 5.5 mM glucose medium; GR Cur, glucose restriction plus curcumin. * *p* < 0.05 vs. control; ***** *p* < 0.0005 vs. control; + *p* < 0.05 vs. GR. **Supplementary Figure S4.** Effect of curcumin and glucose restriction on ATP production in (**A**) Hep3B, (**B**) SNU449, (**C**) HEMa, and (**D**) HDFa cells. Confocal ATP images were taken after 30-min treatment of human melanocytes with curcumin under low-glucose condition (magnification, 400 μL). Con, standard RPMI-1640 medium; GR Cur, glucose restriction plus curcumin. **Supplementary Figure S5.** Confocal imaging of autophagosomes of (**A**) HepG3B and (**B**) HDFa cells. Cells were immunostained with an LC3 antibody. Con, standard RPMI-1640 medium; GR Cur, glucose restriction plus curcumin.

## Abbreviations

CR	calorie restriction
GR	glucose restriction
IGF-1	insulin-like growth factor 1
PI3K	phosphoinositide 3-kinase
mTOR	mammalian target of rapamycin
AMPK	AMP-dependent protein kinase
pHi	intracellular pH
MCT	monocarboxylate transporter
NHE1	Na+/H+ exchanger-1
v-ATPase	vacuolar H+-ATPase
pHe	extracellular pH
mTORC1	mTOR complex-1
4EBP1	4E-binding protein 1
HIF-1	hypoxia inducible factor-1
GTP	guanosine triphosphate
PMA	phorbol-12-myristate-13-acetate
FBS	fetal bovine serum
SDS-PAGE	sodium dodecyl sulfate–polyacrylamide gel electrophoresis
TBS-T	tris-buffered saline with 0.1% Tween 20
PBS	phosphate-buffered saline
BCECF	2’,7’bis-(2-carboxyethyl)-5-(and-6)-carboxyfluorescein acetoxymethyl ester
SEs	standard errors
ANOVA	analysis of variance
