# Supplementary material for: Curcumin Treatment in Combination with Glucose Restriction Inhibits Intracellular Alkalinization and Tumor Growth in Hepatoma Cells
Source: Int J Mol Sci. 2019 May 14;20(10):2375. doi: 10.3390/ijms20102375 (PMC6566721; doi:10.3390/ijms20102375)
Supplement: Supplementary file 1 [file ijms-20-02375-s001.zip › ijms-498547-SI.pptx]

## Slide 1
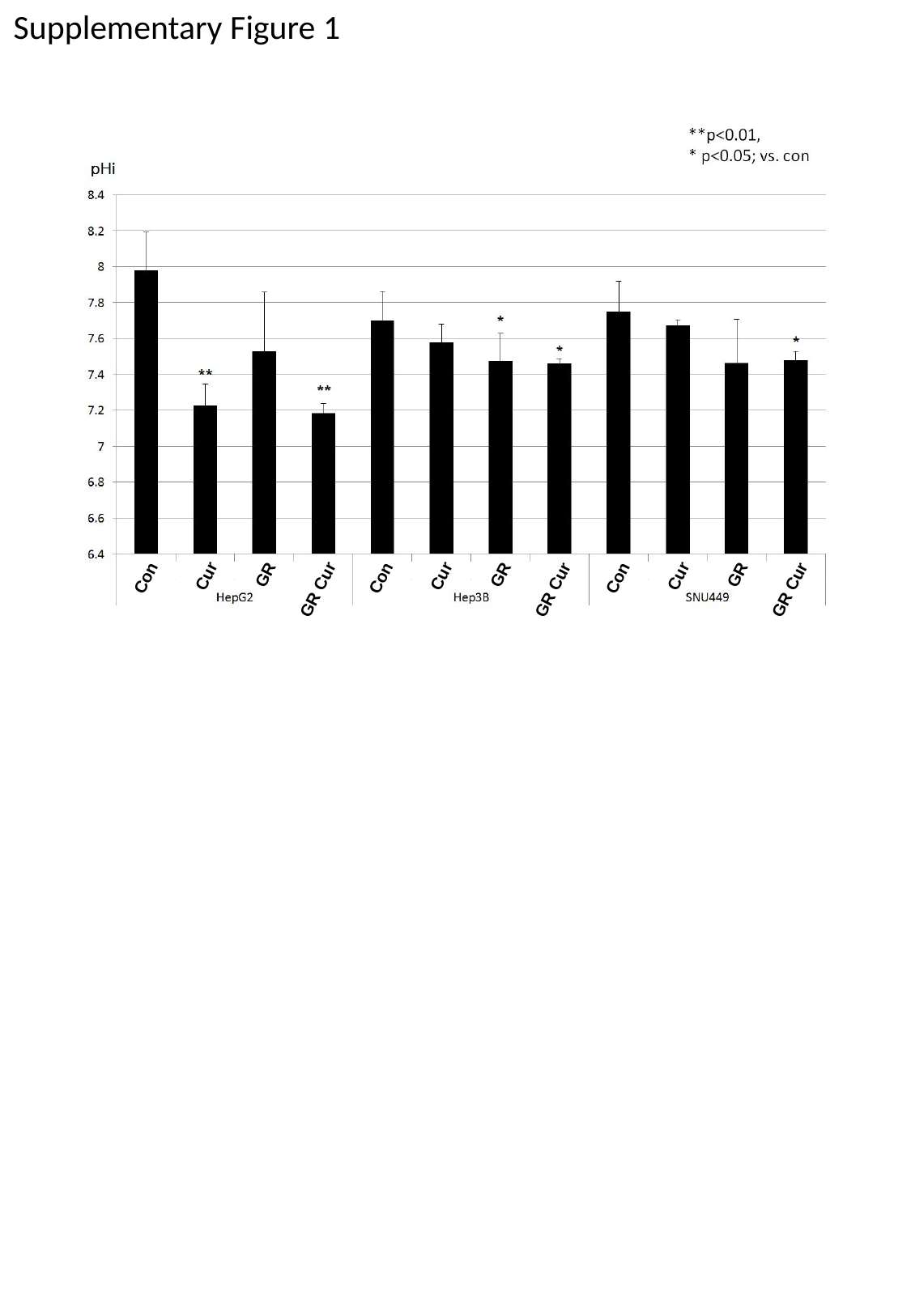

Supplementary Figure 1
Con
Con
Con
Cur
GR
GR Cur
Cur
GR
GR Cur
Cur
GR
GR Cur

## Slide 2
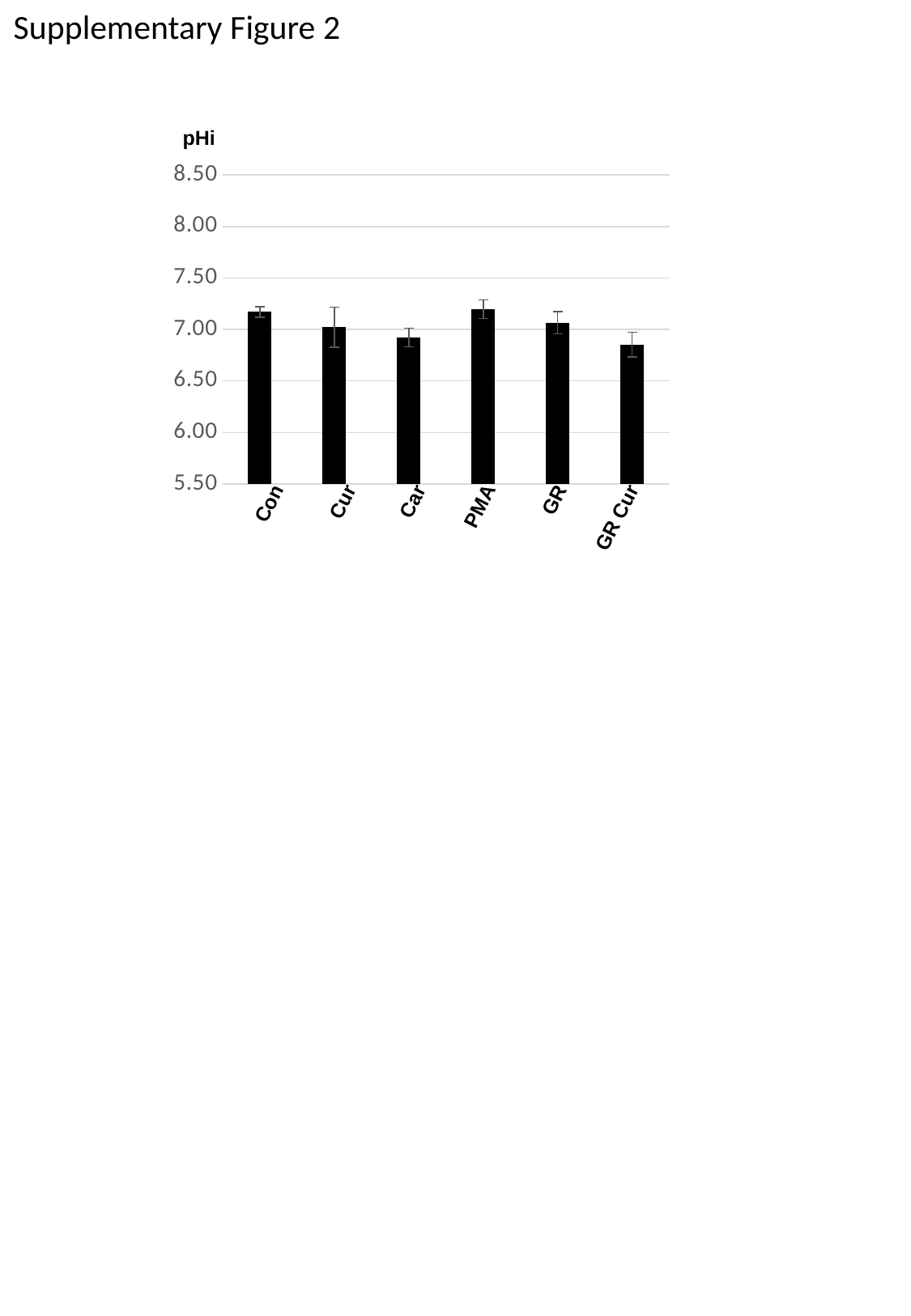

Supplementary Figure 2
pHi
### Chart
| Category | |
|---|---|
| Control | 7.169952560628498 |
| Curcumin | 7.021171253485027 |
| Cariporide | 6.921359814798281 |
| PMA | 7.1974313582645735 |
| Low Glucose | 7.066549896976691 |
| LG+cur | 6.853284656254209 |Car
Con
Cur
PMA
GR
GR Cur

## Slide 3
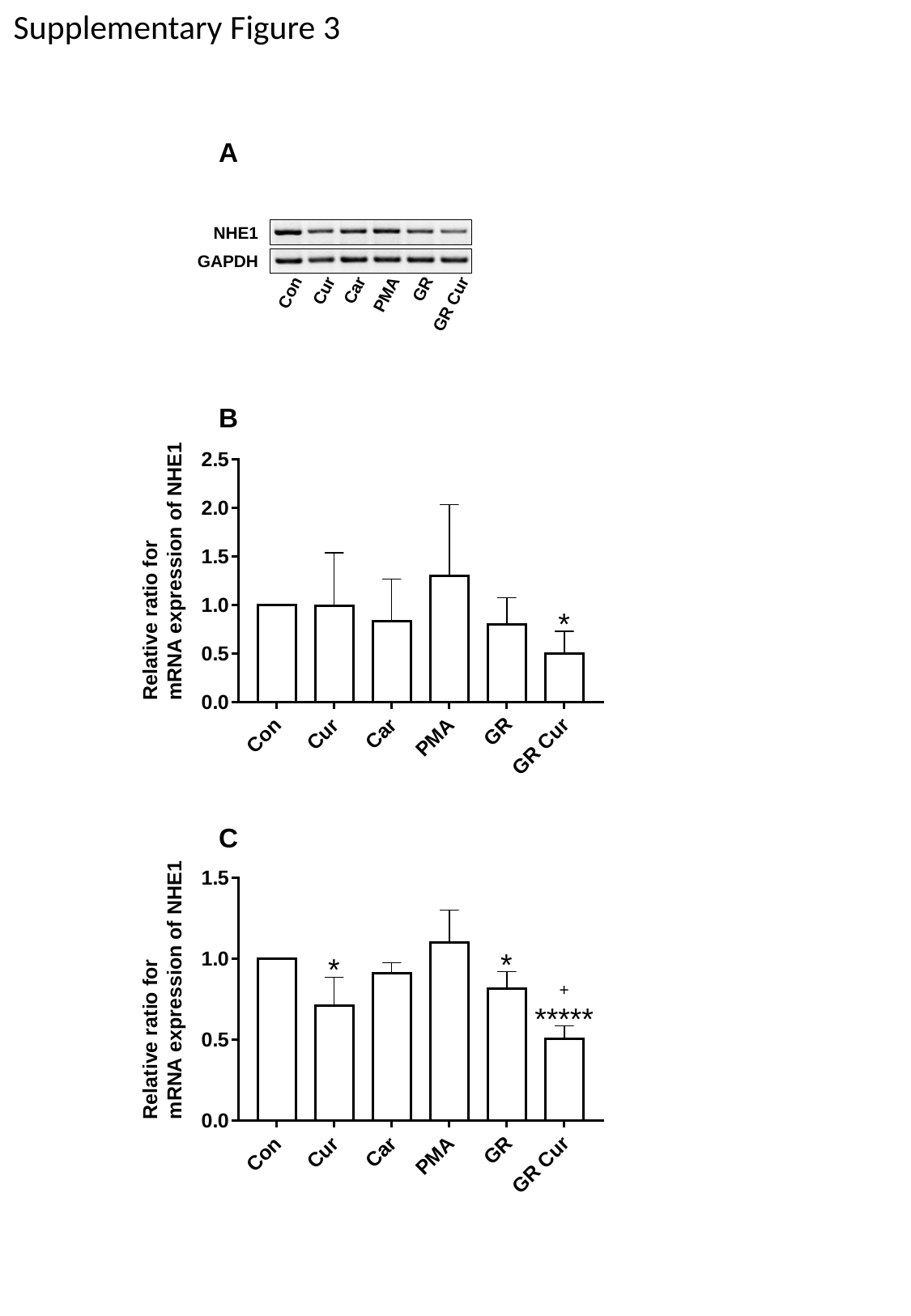

Supplementary Figure 3
A
NHE1
GAPDH
Con
Cur
Car
PMA
GR
GR Cur
B
Relative ratio for
mRNA expression of NHE1
*
C
*
*
Relative ratio for
mRNA expression of NHE1
+
*****

## Slide 4
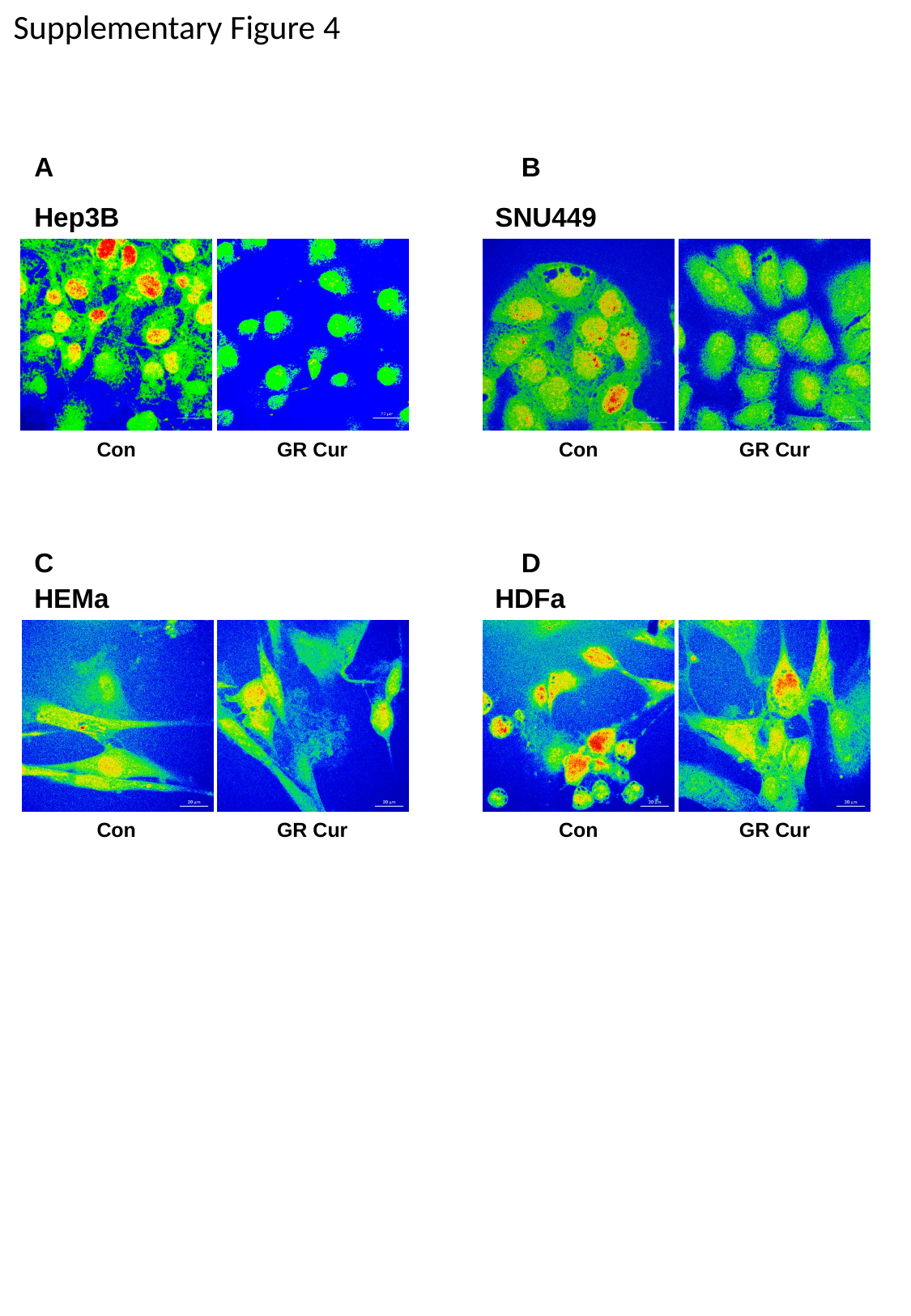

Supplementary Figure 4
A
B
Hep3B
SNU449
GR Cur
GR Cur
Con
Con
C
D
HEMa
HDFa
GR Cur
Con
GR Cur
Con

## Slide 5
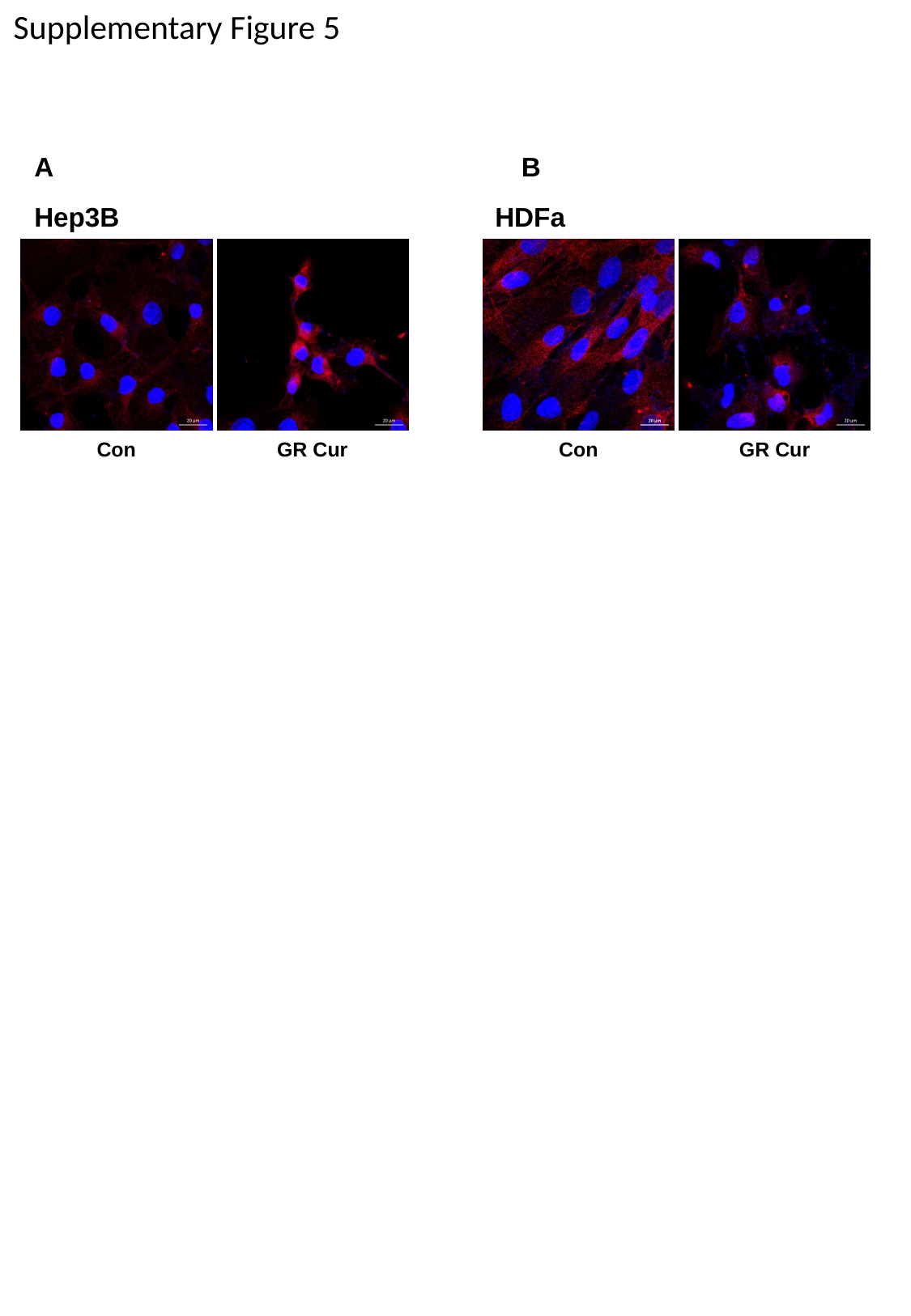

Supplementary Figure 5
A
B
Hep3B
HDFa
GR Cur
GR Cur
Con
Con
